# Transgene *IL-6* Enhances DC-Stimulated CTL Responses by Counteracting *CD4^+^25^+^Foxp3^+^* Regulatory T Cell Suppression via *IL*-*6*-Induced *Foxp3* Downregulation

**DOI:** 10.3390/ijms15045508

**Published:** 2014-03-31

**Authors:** Kalpana Kalyanasundaram Bhanumathy, Bei Zhang, Khawaja Ashfaque Ahmed, Mabood Qureshi, Yufeng Xie, Min Tao, Xin Tan, Jim Xiang

**Affiliations:** 1 Cancer Research Unit, Saskatchewan Cancer Agency, Saskatoon, SK S7N 5E5, Canada; 2 Department of Oncology, University of Saskatchewan, Saskatoon, SK S7N 5E5, Canada; E-Mails: kak677@mail.usask.ca (K.K.B.); bei.zhang@usask.ca (B.Z.); kaa201@mail.usask.ca (K.A.A.); 3 Pathology and Laboratory Medicine, University of Saskatchewan, Saskatoon, SK S7N 5E5, Canada; E-Mail: mabood.qureshi@saskatoonhealthregion.ca; 4 Department of Oncology, the First Affiliated Hospital of Soochow University, Soochow 215000, China; E-Mails: sdxyf@163.com (Y.X.); mtao@medmail.com.cn (M.T.); 5 School of Life Sciences, Beijing Institute of Technology, Beijing 100081, China; E-Mail: tanxing@bit.edu.cn

**Keywords:** dendritic cells (DCs), cytotoxic T lymphocytes (CTLs), interleukin-6 (*IL-6*), forkhead box P3 (*Foxp3*), antitumor immunity

## Abstract

Dendritic cells (DCs), the most potent antigen-presenting cells have been extensively applied in clinical trials for evaluation of antitumor immunity. However, the efficacy of DC-mediated cancer vaccines is still limited as they are unable to sufficiently break the immune tolerance. In this study, we constructed a recombinant adenoviral vector (AdV*_IL-6_*) expressing *IL-6*, and generated *IL-6* transgene-engineered DC vaccine (DC_OVA/_*_IL-6_*) by transfection of murine bone marrow-derived ovalbumin (OVA)-pulsed DCs (DC_OVA_) with AdV*_IL-6_*. We then assessed DC_OVA/_*_IL-6_*-stimulated cytotoxic T-lymphocyte (CTL) responses and antitumor immunity in OVA-specific animal tumor model. We demonstrate that DC_OVA/_*_IL-6_* vaccine up-regulates expression of DC maturation markers, secretes transgene-encoded IL-6, and more efficiently stimulates OVA-specific CTL responses and therapeutic immunity against OVA-expressing B16 melanoma BL6-10_OVA_ *in vivo* than the control DC_OVA/Null_ vaccine. Moreover, DC_OVA/_*_IL-6_*-stimulated CTL responses were relatively maintained in mice with transfer of CD4^+^25^+^*Foxp3^+^* Tr-cells, but significantly reduced when treated with anti-IL-6 antibody. In addition, we demonstrate that IL-6 down-regulates *Foxp3*-expression of CD4^+^25^+^*Foxp3^+^* Tr-cells *in vitro*. Taken together, our results demonstrate that AdV-mediated *IL-6* transgene-engineered DC vaccine stimulates potent CTL responses and antitumor immunity by counteracting CD4^+^25^+^ Tr immunosuppression via IL-6-induced *Foxp3* down-regulation. Thus, *IL-6* may be a good candidate for engineering DCs for cancer immunotherapy.

## 1. Introduction

Immune surveillance by CD8^+^ cytotoxic T lymphocytes (CTLs) represents a major mechanism for the detection and elimination of pathogen-infected cells. CTLs are also essential for effective immunity against tumors [1]. Dendritic cells (DCs) are the most potent professional antigen-presenting cells of the immune system, uniquely capable of stimulating tumor-specific CD4^+^ and CD8^+^ T cell immune responses leading to CTL tumor infiltration and tumor regression [2,3]. DC vaccines have been extensively applied in experimental animal models and clinical trials for evaluation of antitumor immunity [4,5]. While only a proportion of the tumor immunotherapy clinical trials carried out so far have yielded positive results, those using DCs as carrier of tumor antigens have obtained the highest rates of success amongst others [6]. However, in general, the efficacy of DC-mediated cancer vaccine is still limited, mostly because DC vaccines are unable to sufficiently break the suppressive tumor microenvironment and immune tolerance in cancer patients [4].

Inflammatory cytokines such as IL-2, IL-6, IL-12, IL-15 and TNF-α play an important role in inflammation, innate and adaptive immunity [7]. To improve the efficacy of DC vaccine, DCs were genetically modified to produce IL-2 or IL-12 [8,9]. These engineered DC vaccines induced potent antitumor immunity via activation of strong CTL responses. It was also demonstrated that *IL-15* transgene expression of engineered DCs increased their functional effect and survival, and became resistant to tumor-induced DC apoptosis via up-regulation of DC markers and Bcl-2, respectively [10]. We previously demonstrated that inflammatory cytokine *TNF-α* transgene-expressing DCs underwent augmented cellular maturation and induced more robust CTL responses and antitumor immunity [11]. However, the impact of genetically modified-DCs with *IL-6* transgene in antitumor vaccine has not been studied.

In this study, we cloned murine inflammatory cytokine *IL-6* gene from ConA-stimulated T cells by reverse transcription-polymerase chain reaction (RT-PCR) and constructed a recombinant adenoviral vector AdV*_IL-6_* using the cloned *IL-6* cDNA. We then generated *IL-6* transgene-engineered DC (DC*_IL-6_*) vaccine by transfection of murine bone marrow (BM)-derived ovalbumin (OVA)-pulsed DCs (DC_OVA_) with AdV*_IL-6_* and further assessed DC_OVA/_*_IL-6_*-stimulated CTL responses and antitumor immunity in an OVA-specific animal tumor model.

## 2. Results and Discussion

### 2.1. AdV_IL-6_-Transfected DCs Upregulate Expression of I_a_^b^, CD54, CD80 and IL-6

**Figure 1.**
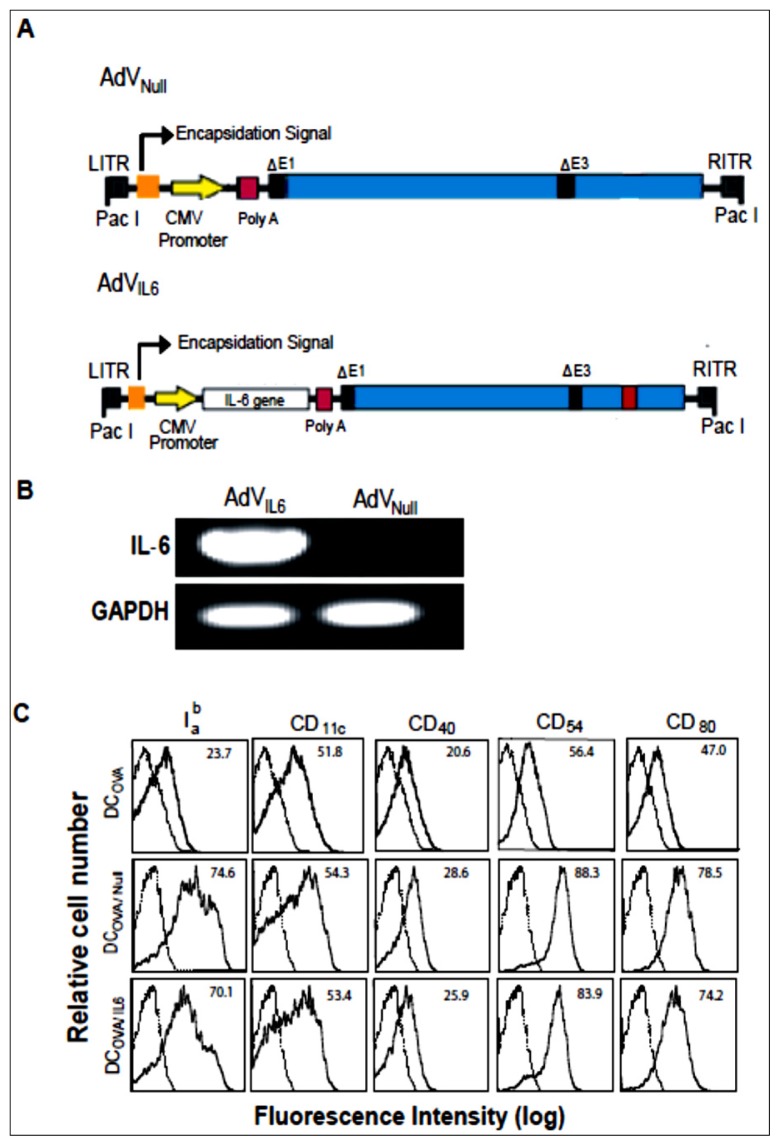
Phenotypic analysis of transgene *IL-6*-engineered DC_OVA/_*_IL-6_* (**A**) Schematic representation of adenoviral (AdV) vector construct expressing *IL-6* gene. The E1/E3 depleted replication-deficient AdV is under the regulation of the cytomegalovirus (CMV) early/immediate promoter/enhancer. ITR, inverted terminal repeat; (**B**) RT-PCR analysis of RNA obtained from AdV*_IL-6_* and AdV_Null_ [*IL-6* Primer sequence: Forward 5′- ACCGC TATGA AGTTC CTCTC TGC -3′; Reverse 5′- AGGCA TAACG CACTA GGTTT GC -3′] [GAPDH Primer sequence: Forward 5′- CAGGT TGTCT CCTGC GACTT -3′; Reverse 5′- CTTGC TCAGT GTCCT TGCTG -3′]; (**C**) AdV transfected DCs were stained with a panel of Abs (solid lines) or isotype-matched control antibodies (dashed lines) followed by flow cytometric analysis. The value in each panel represents the percentage of positive cells based on the isotype control. One representative experiment of two is shown.

To assess the impact of genetically modified-DCs with *IL-6* transgene in antitumor vaccine, we first constructed a recombinant adenoviral vector AdV*_IL-6_* expressing transgene *IL-6* under the regulation of the cytomegalovirus (CMV) early/immediate promoter/enhancer (Figure 1A). To assess the transcriptional *IL-6* expression, RNA extracted from AdV*_IL-6_* was subjected to reverse transcription-polymerase chain reaction (RT-PCR) analysis using glyceraldehyde-3-phosphate dehydrogenase (GAPDH) as the loading control. As shown in Figure 1B, a significant amount of *IL-6* expression was found in recombinant adenovirus AdV*_IL-6_*, but not in the control adenovirus AdV_Null_ without any transgene insertion. We demonstrated that DC_OVA_ expressed DC marker CD11c, adhesion molecule CD54 and DC maturation markers CD40, CD80 and I_a_^b^ (Figure 1C), and secreted little amount of IL-6 (0.03 ng/mL). We also demonstrated that both AdV*_IL-6_*- and AdV_Null_-transfected DC_OVA/_*_IL-6_* and DC_OVA/Null_ up-regulated CD40, CD54, CD80, and I_a_^b^ (Figure 1C), indicating that AdV-mediated transfection enhances DC maturation. In addition, we also found that AdV_Null_-transfected DC_OVA/Null_ secreted some IL-6 (0.40 ng/mL) whereas AdV*_IL-6_*-transfected DC_OVA/_*_IL-6_* secreted much more IL-6 (1.90 ng/mL), indicating that AdV transfection induces DCs to express the inflammatory cytokine IL-6.

### 2.2. AdV_IL-6_-Transfected DCs Stimulate Potent CTL Responses

**Figure 2.**
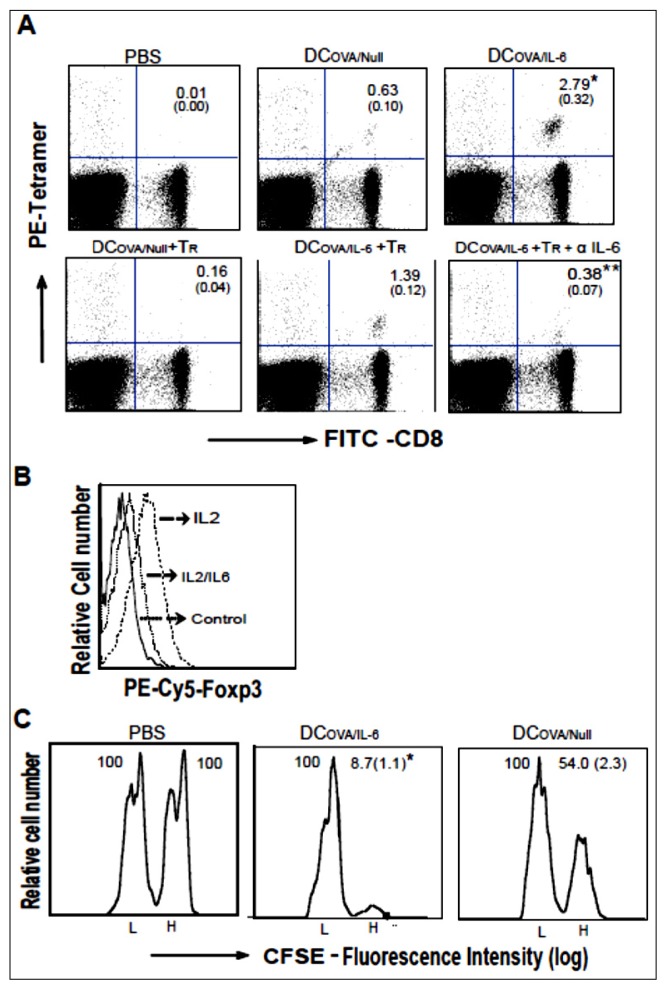
DC_OVA/_*_IL6_* stimulates potent CTL responses. (**A**) C57BL/6 mice were intravenously (i.v.) immunized with PBS, DC_OVA/_*_IL-6_* and DC_OVA/Null_. On day six after immunization, mouse tail blood samples were stained with PE-labeled H-2Kb/OVA_257–264_ tetramer (Beckman-Coulter, Mississauga, ON, Canada) and FITC-labeled anti-CD8^+^ antibody, followed by flow cytometric analysis. One day after CD4^+^25^+^*Foxp3^+^* Tr cells transfer, C57BL/6 mice were i.v. immunized with DC_OVA/_*_IL-6_*, DC_OVA/Null_ and the CTL responses were analyzed by flow cytometry with or without i.v. treatment of anti-IL-6 antibody (0.5 mg/mL). The value in each panel represents the percentage of OVA-specific (tetramer-positive) CD8^+^ T cells *vs.* the total CD8^+^ T cell population. The value in parenthesis represents the standard deviation (SD). * *p* < 0.05 *vs.* cohorts of the DC_OVA/Null_ group and ** *p* < 0.05 *vs.* cohorts of DC_OVA/_*_IL-6_* + Tr group (student *t* test); (**B**) CD4^+^25^+^*Foxp3^+^* Tr cells were incubated with IL-2 with or without IL-6 overnight. After fixation, the cell membranes were permeabilized and then stained with PE-Cy5-conjugated anti-Foxp3^+^ antibody followed by flow cytometric analysis; (**C**) *In vivo* cytotoxicity assay. Six days after immunization, the immunized mice were i.v. injected with a mixture of CFSE^high^ and CFSE^low^-labeled splenocytes (at 1:1 ratio) that had been pulsed with OVAI and the control Mut1 peptide, respectively. After sixteen hours, spleens of immunized mice were removed and the percentages of the residual CFSE^high^ (H) and CFSE^low^ (L) target cells remaining in the recipients’ spleens were analyzed by flow cytometry. The value in each panel represents the percentage of CFSE^high^ *vs.* CFSE^low^ target cells remaining in spleen. The value in parenthesis represents the standard deviation (SD). * *p* < 0.05 *vs.* cohorts of the DC_OVA/Null_ group (student *t* test). One representative experiment of two is shown.

To assess DC_OVA/_*_IL-6_* vaccine-stimulated CTL responses, we intravenously (i.v.) immunized C57BL/6 mice with DC_OVA/_*_IL-6_*. Six days later, the amount of OVA-specific CD8^+^ T cells in the peripheral blood was measured using PE-labeled H-2K^b^/OVA_257–264_ tetramer and FITC-anti-CD8^+^ antibody staining by flow cytometry. As illustrated in Figure 2A, the percentage of double positive (PE-tetramer^+^ and FITC-CD8^+^) cells in the total CD8^+^ population is significantly higher in the DC_OVA/_*_IL-6_*-immunized mice (2.79%) compared to the control DC_OVA/Null_-immunized mice (0.63%) (*p* < 0.05), with both immunized groups showing a significant difference compared to the control PBS-immunized mice (*p* < 0.05), indicating that DC_OVA/_*_IL-6_* immunization stimulates potent OVA-specific CD8^+^ T cell responses.

### 2.3. AdV_IL-6_-Transfected DCs Counteract CD4^+^25^+^Foxp3^+^ Tr Immunosuppression via Transgene Encoded IL-6 Signaling

To assess the potential counteraction of CD4^+^25^+^*Foxp3^+^* Tr immunosuppression, the CTL responses of immunized C57BL/6 mice, previously infused with naïve CD4^+^25^+^*Foxp3^+^* Tr cells, were assessed by flow cytometry. We demonstrated that DC_OVA/Null_-stimulated CTL responses were significantly decreased (0.16%) in mice infused with CD4^+^25^+^*Foxp3^+^* Tr cells (*p* < 0.05), whereas DC_OVA/_*_IL-6_*-stimulated CTL responses were relatively maintained (1.39%) in CD4^+^25^+^*Foxp3^+^* Tr-infused mice (Figure 2A), indicating that DC_OVA/_*_IL-6_* vaccine counteracts CD4^+^25^+^*Foxp3^+^* Tr immunosuppression. To confirm it, we also blocked IL-6 signaling in DC_OVA/_*_IL-6_*-vaccinated mice by anti-IL-6 antibody treatment. We found that DC_OVA/_*_IL-6_*-stimulated CTL responses became significantly reduced (*p* < 0.05) in anti-IL-6 antibody-treated mice (Figure 2A), suggesting that DC_OVA/IL-6_ vaccine counteracts CD4^+^25^+^*Foxp3^+^* Tr immunosuppression possibly via transgene-encoded *IL-6* signaling.

### 2.4. IL-6 Induces Foxp3 down-Regulation of CD4^+^25^+^Foxp3^+^ Tr Cells

IL-6 has been reported to inhibit the generation and counteract the immunosuppression of CD4^+^25^+^*Foxp3^+^* Tr cells [12,13]. To assess the mechanism for IL-6-induced counteraction, we cultured CD4^+^25^+^*Foxp3^+^* Tr cells in the presence or absence of IL-6. We found that IL-6-treated CD4^+^25^+^*Foxp3^+^* Tr cells down-regulated *Foxp3* expression (Figure 2B), indicating that IL-6-induced counteraction of CD4^+^25^+^*Foxp3^+^* Tr immunosuppression may be via *Foxp3* down-regulation.

### 2.5. AdV_IL-6_-Transfected DC-Stimulated CD8^+^ T Cells Are Effector CTLs

To analyze the differentiation of DC_OVA/_*_IL-6_***-**stimulated CD8^+^ T cells into effector CTLs, an *in vivo* cytotoxicity assay was performed. We adoptively i.v. transferred OVAI peptide-pulsed and strongly carboxyfluorescein diacetate succinimidyl ester (CFSE)-labeled splenocytes (CFSE^high^) as the OVA-specific target cells, as well as the control peptide-pulsed and weakly CFSE-labeled splenocytes (CFSE^low^) as the control non-specific target cells into recipient mice six days after immunization with DC_OVA/_*_IL-6_* and DC_OVA/Null_. Flow cytometric analysis was performed to examine the ability of activated T cells to induce specific killing of the above target cells sixteen hours after target cell transfer. In Figure 2C, the cell killing was specifically targeted towards OVAI-pulsed CFSE^high^ target cells, and the levels of CFSE^low^ cells remain unaffected. Mice immunized with DC_OVA/Null_ had a decrease of 46% OVAI-pulsed CFSE^high^ target cells, whereas mice immunized with DC_OVA/_*_IL-6_* had a significantly greater degree of loss of OVAI-pulsed CFSE^high^ target cells (91.3%) (*p* < 0.05), indicating that DC_OVA/_*_IL-6_*-stimulated CD8^+^ T cells are effector CTLs with more efficient killing activity for OVA-specific target cells.

### 2.6. AdV_IL-6_-Transfected DCs Induce Potent Antitumor Immunity

To study whether DC_OVA/_*_IL-6_* is capable of inducing therapeutic immunity against six-day-established tumor, we i.v. injected mice with the highly metastatic OVA-expressing B16 melanoma cells BL6-10_OVA_ (1 × 10^6^ cells). Six days later, mice were i.v. immunized with DC_OVA/_*_IL-6_*. Three weeks after tumor cell challenge, mice were sacrificed and numbers of lung metastatic tumor colonies were counted. As shown in Table 1, DC_OVA/Null_ immunization only cured 50% of the mice (4/8). However, the median number of lung tumor colonies in DC_OVA/Null_-immunized group was 49, which is much less than that (>300) in PBS control group (*p* < 0.05). In contrast, DC_OVA/_*_IL-6_* immunization was able to protect 100% of mice (8/8) from tumor growth, indicating that DC_OVA/_*_IL-6_* vaccine stimulates potent therapeutic immunity against six-day-established B16 melanoma.

### 2.7. Discussion

Dendritic cells (DCs) are a subset of white blood cells that are critical to most aspects of adaptive immunity because of their central role in initiation of T-cell responses [14–16]. As dendritic cells (DCs) are the most potent antigen-presenting cells (APCs) [14–16], engineering DCs is likely to yield improved therapeutic vaccines [17] by inducing or promoting efficient antitumor immune responses in cancer patients [18]. Previous reports indicate that intratumoral injection of DCs, engineered to express a combination of different cytokines, such as IL-12, IL-21, or IFN-α, showed potent therapeutic effect against established tumors [19]. Vogt *et al.* [20] have reported that intratumoral injection of adenoviral vector-transfected DCs with *IL-12* over-expression was crucial for effective tumor regression. Qu *et al.* [21] have demonstrated the therapeutic effectiveness of intratumorally delivered DCs engineered to express the pro-inflammatory cytokine IL-32.

The cytokine IL-6 secreted by many different cells, including the monocyte/macrophages, fibroblasts, endothelial cells, keratinocytes, mast cells, T cells, and DCs acts as a central regulator of inflammatory processes [22]. It plays a key role in progression from the initial innate immune responses to infection to adaptive immune responses [23]. IL-6 is involved in the maturation of B cells and development of a major proinflammatory T cell population, the pathogenic CD4^+^ Th17 cells [24,25]. IL-6 has been reported to inhibit the generation of and counteract the immunosuppression of CD4^+^25^+^ Tr cells [12,13]. We have shown that IL-6 counteracts CD4^+^ Th2 cell’s IL-10-mediated immunosuppression [26]. These unique characteristics of IL-6 suggest that it may be a good candidate transgene to engineer DCs for the development of new DC-based vaccines capable of overcoming immunosuppression leading to potent CTL responses and antitumor immunity.

To assess the impact of genetically modified-DCs with *IL-6* transgene in antitumor vaccine, we constructed the recombinant adenoviral vector AdV*_IL-6_* expressing transgene *IL-6* and the control adenoviral vector AdV_Null_ without any transgene insertion. We have previously shown that AdV transfected DCs expressed inflammatory cytokines such as IL-1β and IL-12 [27]. In this study, we found that AdV_Null_-transfected DC_OVA/Null_ secreted IL-6 (0.4 ng/mL), indicating that AdV transfection also induces DCs to express inflammatory cytokine IL-6. Furthermore, AdV*_IL-6_***-**transfected DC_OVA/_*_IL-6_* secreted much more IL-6 (1.95 ng/mL), indicating that DC_OVA/_*_IL-6_* cells also secrete transgene-encoded IL-6. In addition, we also demonstrated that both AdV*_IL-6_*- and AdV_Null_**-**transfected DC_OVA/_*_IL-6_* and DC_OVA/Null_ up-regulated CD54, CD80 and I_a_^b^, indicating that AdV-mediated transfection enhances DC maturation, which is consistent with several previous reports [27–29]. The AdV**-**induced DC maturation has been shown to be linked to *NF-κB*-dependent [30] and PI3 kinase-mediated *TNF-α* induction pathway [31].

In this study, we demonstrated that DC_OVA/_*_IL-6_* vaccine stimulates potent effector CTL responses and immunity against OVA-expressing B16 melanoma. The polyclonal naïve CD4^+^25^+^*Foxp3^+^* Tr cells develop in the thymus and then enter peripheral tissues where they suppress the activation of other self-reactive T cells [32]. The transcription factor Foxp3 controls regulatory T cell development [33,34]. The activation and transgene expression of *Foxp3* have been reported to induce immune suppressive effects of T cells, DCs and macrophages [35,36]. It has also been shown that an elevated number of Tr cells was detected in tumors [37,38], which suppressed the antitumor immune responses by inhibition of T cell proliferation and effector function [39–41] as well as DC maturation [42]. Therefore, the question of how to combat immune tolerance becomes a critical challenge in cancer vaccine development [43]. In this study, we demonstrate that DC_OVA/_*_IL-6_* vaccine counteracts CD4^+^25^+^*Foxp3^+^* Tr-mediated immunosuppression in mice with transfer of purified naïve CD4^+^25^+^*Foxp3^+^* Tr cells. In addition, for the first time, we demonstrate that the counteraction of CD4^+^25^+^*Foxp3^+^* Tr suppression by DC_OVA/_*_IL-6_* vaccine is derived from transgene-encoded *IL-6* signaling and possible via IL-6-induced *Foxp3* down-regulation. OVA protein is a well-established model antigen to study anti-tumor immunity [44]. Many previous studies also have used OVA as a model antigen for tumor immunotherapeutic studies [45–47]. However, future study using less immunogenic tumor antigen will be interesting. Overall, our results suggest that IL-6 may overcome Tr-mediated suppression of antigen-specific T cell responses in tumor microenvironments. It has also been demonstrated that IL-6 activates *in vivo* T cell responses [12] and exerts anti-apoptotic activity on a wild variety of cells, including the naïve and activated T cells [48–50]. Therefore, the potent CTL responses and antitumor immunity induced by DC_OVA/_*_IL-6_* vaccine may be derived from a combination of the above transgene-encoded IL-6-mediated stimulatory effects.

## 3. Experimental Section

### 3.1. Reagents, Cell Lines and Animals

The biotin-labeled antibodies (Abs) specific for CD11c, CD40, CD54, CD80, I_a_^b^, and fluorescein isothiocyanate (FITC)- or phycoerythrin (PE)-labeled Abs specific for CD4, CD8, and CD44 were obtained from PharMingen Canada Inc. (Mississauga, ON, Canada). The anti-ovalbumin (OVA) Ab was obtained from Sigma (Oakville, ON, Canada). The PE-labeled H-2K^b^/OVA_257–264_ tetramer was obtained from Beckman Coulter (San Diego, CA, USA). The PE-Cy5-conjugated anti mouse Foxp3 antibody was obtained from eBioscience Inc. (San Diego, CA, USA). The highly lung metastatic OVA-expressing B16 melanoma cell line BL6-10_OVA_ was generated in our laboratory [51]. Naïve C57BL/6 mice were obtained from Jackson Laboratory (Bar Harbor, ME, USA). All animal experiments were carried out in accordance with the Canadian Council for Animal Care guidelines.

### 3.2. Recombinant Adenovirus Construction

The construction of recombinant adenovirus (AdV) expressing *IL-6* (AdV*_IL-6_*) was performed by insertion of mouse *IL-6* gene cloned from ConA-stimulated T cells into pShuttle vector (Stratagene Inc., La Jolla, CA, USA) using the cloned *IL-6* cDNA to form pLpA*_IL-6_* expressing *IL-6* gene [11]. The PmeI-digested shuttle vector was then co-transformed into BJ5183 *E. coli* cells already containing the backbone vector for homologous recombination to form the recombinant vector AdV*_IL-6_* as described previously (Figure 1A) [52,53]. The control AdV_Null_ without any transgene insert was previously constructed in our laboratory (Figure 1A) [52,53]. The recombinant AdV*_IL-6_* vector was then linearized by PacI digestion, and then transfected into 293 cells using lipofectamine (Gibco/BRL, Burlington, ON, Canada) to generate recombinant adenovirus AdV*_IL-6_* as described previously [52,53]. All recombinant AdVs were amplified in 293 cells and purified by cesium chloride ultracentrifugation gradients [52,53]. To assess transgene *IL-6* expression, we performed RT-PCR using RNA purified from AdV*_IL-6_*-transfected 293 cells as described previously [52,53].

### 3.3. Preparation of Dendritic Cells

C57BL/6 mouse bone marrow (BM)-derived dendritic cells (DCs) were prepared as described previously [1]. Briefly, BM cells from femora and tibia of naïve C57BL/6 mice were depleted of red blood cells with 0.84% Tris-ammonium chloride, and plated in DC culture medium (Dulbecco’s Modified Eagle Medium (DMEM) plus 10% fetal calf serum (FCS), granulocyte macrophage colony-stimulation factor (GM-CSF) (20 ng/mL) and IL-4 (20 ng/mL)). On day three, the non-adherent granulocytes, T and B cells were gently removed and fresh media was added. After two days, the loosely adherent proliferating DC aggregates were dislodged and re-plated. On day six, DCs displaying typical morphologic features (that is, numerous dendritic processes) were further pulsed with ovalbumin (OVA) (0.5 mg/mL) for overnight and termed DC_OVA_.

### 3.4. Preparation of CD4^+^25^+^Foxp3^+^ Regulatory T (Tr) Cells

Mouse splenocytes were first depleted of red cells with 0.84% Tris-ammonium chloride. T cells were purified by passing splenocytes through nylon wool-columns as described previously [1]. Naïve CD4^+^ T cells were purified by using Dynal CD8 microbeads (Dynal Inc., Lake Success, NY, USA), and CD4^+^25^+^*Foxp3^+^* Tr cells were then further purified from naïve CD4^+^ T cell population using biotin-anti-CD25 antibody and anti-biotin MACS beads (Miltenyi Biotech, Auburn, CA, USA), as previously described [54].

### 3.5. Adenovirus (AdV) Transfection of DCs

DC_OVA_ were transfected with AdV*_IL-6_* expressing the transgene *IL-6* and control AdV_Null_ without any transgene insert at a multiplicity of infection (MOI) of 150 to form DC_OVA/_*_IL-6_* and DC_OVA/Null_ vaccines as previously described [52,53]. The transfected cells were then harvested for phenotypic analysis by flow cytometry. Moreover, the supernatants of DC_OVA/_*_IL-6_* and DC_OVA/Null_ were assessed for the secretion of IL-6 using the IL-6 enzyme-linked immunosorbent assay (ELISA) kit (BD Bioscience, Mississauga, ON, Canada).

### 3.6. Flow Cytometric Analysis

For phenotypic analysis, DC_OVA_, DC_OVA/_*_IL-6_*, and DC_OVA/Null_ were stained with biotin-conjugated anti-mouse antibodies (2 mg/mL) specific for major histocompatibility complex (MHC) class II (I_a_^b^), CD40, CD54 or CD80 and the cells were analyzed by flow cytometry. For tetramer analysis, peripheral blood of immunized C57BL/6 mice [DC_OVA_, DC_OVA/_*_IL-6_*, and DC_OVA/Null_ (1 × 10^6^ cells/mouse)] were stained with PE-labeled H-2K^b^/OVA_257–264_ tetramer and FITC-labeled anti-CD8 antibody on day six after immunization followed by flow cytometric analysis. In another set of experiments, mice were first i.v. transferred with CD4^+^25^+^*Foxp3^+^* Tr cells (1 × 10^6^ cells/mouse). One day after Tr cell transfer, mice were i.v. immunized with DC_OVA/_*_IL-6_*, DC_OVA/Null_ with or without i.v. treatment of anti-IL-6 antibody (0.5 mg/mL), and the CTL responses were analyzed six days after immunization by flow cytometry. To assess *Foxp3* expression, CD4^+^25^+^*Foxp3^+^* Tr cells were incubated in culture medium with IL-2 (40 units/mL) in the presence or absence of IL-6 (40 ng/mL) for overnight. The cells were permeabilized with cytofix/cytoperm solution (BD Biosciences, San Diego, CA, USA) and then stained with PE-Cy5-conjugated anti-Foxp3 antibody followed by flow cytometric analysis.

### 3.7. In Vivo Cytotoxicity Assay

The *in vivo* cytotoxicity assay was performed as described previously [51]. Briefly splenocytes derived from naïve C57BL/6 mice were incubated with high (3.0 μM, CFSE^high^) or low (0.6 μM, CFSE^low^) concentrations of CFSE. CFSE^high^ cells were further pulsed with OVAI (OVA_257–264_) peptide (SIINFEKL), and washed extensively to remove free peptide. However, the CFSE^low^ cells were pulsed with the control Mut peptide (FEQNTAQP) to become the internal controls. CFSE^high^ and CFSE^low^ target cells were co-injected i.v. at a ratio of 1:1 into the above immunized mice six days after immunization. Sixteen hours after injection, spleens were removed from the immunized mice to analyze the residual OVA-specific CFSE^high^ and irrelevant control CFSE^low^ target cells remaining in recipients’ spleens by flow cytometry.

### 3.8. Animal Studies

For evaluation of therapeutic antitumor immunity, C57BL/6 mice were first challenged by i.v. injection with 1 × 10^6^ OVA-expressing BL6-10_OVA_ tumor cells. Six days after tumor cell injection, mice were vaccinated i.v. with 1 × 10^6^ engineered DC_OVA/IL-6_, DC_OVA/Null_, and PBS, respectively. Three weeks after tumor cell challenge, mice were sacrificed, and numbers of lung metastatic tumor colonies were counted. The metastasis on freshly isolated lungs appeared as discrete black pigmented foci that can be easily distinguishable from normal lung tissues and further confirmed by histopathological examination. Tumor metastatic foci too numerous to count were assigned an arbitrary value of >300.

### 3.9. Statistical Analyses

Statistical analyses were performed using Student’s *t*-test or Mann-Whitney *U* test to compare variables from different groups [54]. A value of *p* < 0.05 is considered significant.

## 4. Conclusions

The results of our study demonstrate that AdV-mediated *IL-6* transgene-engineered DC vaccine stimulates potent CTL responses and antitumor immunity by counteracting CD4^+^25^+^ Tr immunosuppression via IL-6-induced *Foxp3* down-regulation. Thus, *IL-6* may be a good candidate for engineering DCs for effective cancer immunotherapy.

## Figures and Tables

**Table 1.** DC_OVA/_*_IL6_* induces therapeutic antitumor immunity.

Animal groups	Tumor cell challenge	Tumor bearing mice (%)	Median number of lung tumor colonies
DC_OVA/Null_	BL6-10_OVA_	4/8 (50)	49 ± 13 *
DC_OVA/IL-6_	BL6-10_OVA_	0/8 (0)	0
PBS	BL6-10_OVA_	8/8 (100)	>300

C57BL/6 mice were i.v. injected with 1 × 10^6^ OVA-expressing BL6-10_OVA_ tumor cells. Six days after tumor cell injection, mice were i.v. immunized with engineered DC_OVA/_*_IL-6_*, DC_OVA/Null_, and PBS, respectively. Three weeks after tumor cell challenge, mice were sacrificed and the numbers of lung metastatic tumor colonies were counted.

* *p* < 0.01 *vs.* cohorts of the DC_OVA/_*_IL-6_* and PBS groups (Mann-Whitney *U* test). One representative experiment of three is shown.
